# Phenotypic characteristics of paroxysmal dyskinesia in 25 cats

**DOI:** 10.1111/jsap.70125

**Published:** 2026-03-25

**Authors:** T. Liatis, C. Maeso, A. De Stefani, A. Pergande, T. Elvira, A. Suñol

**Affiliations:** ^1^ Queen Mother Hospital for Animals Royal Veterinary College London UK; ^2^ Plakentia Veterinary Clinic – Alimos, Alimos Athens Greece; ^3^ School of Veterinary Medicine European University Cyprus Nicosia Cyprus; ^4^ AniCura Ars Veterinaria Hospital Veterinari Barcelona Spain; ^5^ Southern Counties Veterinary Specialists, Part of IVC Evidensia Limited Ringwood UK; ^6^ Anderson Moores Veterinary Specialists, Part of Linnaeus Veterinary Limited Hursley UK; ^7^ Department of Veterinary Medicine, Faculty of Biomedical and Health Sciences Universidad Europea de Madrid Madrid Spain; ^8^ Hospital Veterinaria del Mar, IVC Evidensia Barcelona Spain

## Abstract

**Objectives:**

Paroxysmal dyskinesia has, to date, been reported only systematically in Sphynx cats. This study aims to describe paroxysmal dyskinesia in additional breeds.

**Materials and Methods:**

The medical records of cats from multiple hospitals presenting with episodes consistent with paroxysmal dyskinesia between 2020 and 2025 were retrospectively reviewed, and those with available video footage at presentation were included.

**Results:**

Twenty‐five cats were included. Domestic shorthair cats (11/25; 44%) were overrepresented. Median age at presentation was 3.5 years (range: 1 to 14 years). During an episode, cats were upright standing (14/25; 56%) or crawling (11/25; 44%). Most common signs were shifting limb dystonia (25/25; 100%), bradykinesia (slow‐motion movement) (11/25; 44%), abnormal gait (11/25; 44%), kyphosis (10/25; 40%), tail dystonia (10/25; 40%), cervical dystonia (9/25; 36%) with dystonic head tremor (3/25; 12%), falling (7/25; 28%) or ‘kneading’ digits (5/25; 20%). Neurological examination was normal in all cats. Treatment was attempted in 11/25 (44%) cats with a gluten‐free diet trial (6/11; 55%). Gluten‐free diet trial (4/6) and levetiracetam (2/2) decreased episodes of paroxysmal dyskinesia. Most cats (21/25; 84%) were presumptively diagnosed with idiopathic paroxysmal non‐kinesigenic dyskinesia, whereas 4/25 (16%) were diagnosed with presumptive paroxysmal gluten‐sensitive dyskinesia.

**Clinical Significance:**

Idiopathic paroxysmal dyskinesia occurs in multiple cat breeds and has some distinct clinical features compared to dogs such as crawling, crossing of the thoracic limbs resembling proprioceptive ataxia, bradykinesia (slow‐motion movements), ‘kneading’ of the digits or tail dystonia.

## INTRODUCTION

Paroxysmal dyskinesia (PxD) is a group of involuntary, hyperkinetic, self‐limiting movement disorders that recur episodically and may last from seconds to hours (Mandigers et al., 2024). Dystonia is the most characteristic sign of PxD, and it is characterised by sustained or intermittent involuntary muscle contractions causing abnormal (often repetitive) movements, postures or both (Santifort & Mandigers, 2022). Paroxysmal dyskinesia has been widely reported in dogs, and it is classified as (1) paroxysmal kinesigenic dyskinesia that initiates after sudden movements, (2) paroxysmal non‐kinesigenic dyskinesia, which can occur at rest, and (3) paroxysmal exertion‐induced dyskinesia which is associated with fatigue (Mandigers et al., 2024). The most common cause of PxD is idiopathic (presumptive hereditary); however, reactive, dietary or structural cases have also been reported in dogs (Mandigers et al., 2024).

To date, idiopathic PxD has been described in Sphynx cats, considered to be kinesigenic (James et al., 2022), and one DSH cat (Maeso & Morales, 2020), and recently PxD associated with hyperthyroidism in seven cats (Espinosa et al., 2026). The aim of this study is to describe the clinical phenotype, clinical and diagnostic findings, treatment and outcome of PxD in multiple cat breeds.

## MATERIALS AND METHODS

This was a retrospective, observational, multicentric, case‐series study conducted by four veterinary referral hospitals between January 2020 and March 2025. Search terms included: dyskinesia and cat or feline. Inclusion criteria consisted of (1) complete medical records including a complete neurological examination, (2) cats presented with paroxysmal episodes consistent with dystonia (Cerda‐Gonzalez et al., 2001) based on video footage provided at the time of presentation and (3) a presumptive diagnosis of PxD.

Complete medical records consisted of signalment, presenting complaints, clinical and neurological findings, paroxysmal episode features, clinicopathological and diagnostic imaging findings, treatment and outcome. Assessment of paroxysmal episode features was performed based on detailed clinical description (based on history and video assessment at the time of presentation) and retrospective evaluation of video footage that was reviewed at the time of data collection. All neurological examinations were performed by a board‐certified neurologist or a neurology resident under the direct supervision of a board‐certified neurologist. MRI, CT, cerebrospinal fluid (CSF) analysis findings and other diagnostic test results were evaluated when available. Magnetic resonance imaging devices used included high‐field or low‐field magnets (Intera 1.5T, Philips Healthcare, Amsterdam, Netherlands; 1.5T, Vantage Elan, Canon Medical Systems; 1.5T, General Electric Healthcare Systems, UK; Petvet Hallmarq, Surrey, UK). Magnetic resonance imaging sequences performed varied between institutions but always included transverse and sagittal T2‐weighted (T2W), transverse fluid attenuation inversion recovery and transverse and sagittal T1‐weighted (T1W) pre‐ and post‐contrast (gadopentetate dimeglumine 0.1 mmol/kg IV bolus) images. Follow‐up information was obtained *via* data collection from discharge notes, clinical re‐examinations, phone call or email communication log.

Descriptive statistical analysis was performed using standard statistical software (SPSS Statistics 26, IBM Corporation, Armonk, New York). Data were assessed for normal distribution using the Shapiro–Wilk test for normality. Numerical variables abnormally distributed were presented as median, interquartile range (IQR) and range. Categorical variables were summarised as counts and percentages.

## RESULTS

Twenty‐five cats met the inclusion criteria. Breeds included domestic shorthair cat (11/25; 44%), which was overrepresented, followed by domestic longhair cat (4/25; 16%), Bengal (3/25; 12%), Sphynx (3/25; 12%), Ragdoll (2/25; 8%), Cornish Rex (1/25; 4%) and Nebelung (1/25; 4%). Only 10/25 (40%) of the cats were purebred. Fourteen (56%) cats were male and 11/26 (44%) were female; most cats (19/25; 76%) were neutered. Median age at presentation was 3.5 years (range: 1 to 14 years; interquartile range [IQR]: 12 years). Median body weight was 3.4 kg (range: 3 to 7 kg; IQR: 4 kg).

All cats presented because of paroxysmal episodes. A minority of cats (2/25; 8%) had accompanying gastrointestinal signs such as diarrhoea or vomiting. Median duration between first episode and presentation was 21 days (range: 0 to 45 days; IQR: 26 days). Progressive frequency of abnormal episodes since onset occurred in 11/25 (44%), whereas in 6/25 (24%) cats, episodes manifested in clusters with long episodes of paucity (*e.g*. multiple episodes in a week or a month and then paucity for 6 months). In the remaining cats, 7/25 (28%), the episode frequency was sporadic without progression or cluster periods. Median duration of the paroxysmal episodes was 5 minutes (range: 0.5 to 32 minutes; IQR: 3.45 minutes), whereas the frequency of episodes varied from multiple per day to sporadic per year. The owners reported presumptive triggers in some cats such as anxiety (5/25; 20%), dietary change (1/25; 4%) or excitement (1/25; 4%). There was no report of sudden movement as a trigger for an episode in any cat. Anxiety was reported by the owners within the history as a general lifestyle characteristic of 6/25 (24%) cats.

Video footage was available at the time of presentation for all cats, whereas video footage from 19/25 (76%) cats was available for review at the time of writing the study (Video 1). Posture during an episode was upright standing (14/25; 56%) or crawling posture or gait (11/25; 44%). Shifting limb dystonia was the hallmark of semiology during an episode in all cats (25/25; 100%), followed by bradykinesia (slow‐motion movement) (11/25; 44%), abnormal gait manifested as involuntary crossing of thoracic limbs which was perceived as proprioceptive ataxia (11/25; 44%), kyphosis (10/25; 40%), tail dystonia including flexion or upward extension of the tail (10/25; 40%), cervical dystonia (9/25; 36%) with dystonic head tremor (3/25; 12%), falling (7/25; 28%), ‘kneading’ digits (5/25; 20%), mydriasis (4/25; 16%), truncal sway (4/25; 16%), head sway (3/25; 12%) and lip smacking (3/25; 12%). No autonomic signs such as hypersalivation, urination or defecation were reported by the owners. All cats were reported to be responsive during the episodes, and they were normal in between the episodes.

**Video 1:** Semiology of paroxysmal dyskinesia in cats.

Neurological examination was normal in all cats but one, which was consistent with pelvic limb proprioceptive ataxia and ambulatory paraparesis with normal postural reactions and spinal reflexes. Neuroanatomical localisation was forebrain (basal nuclei) in all cats but one, which also involved concurrent T3–L3 spinal cord segments (the cat with paraparesis and pelvic limb proprioceptive ataxia).

Haematology was within normal limits in 22/25 (88%) cats, and consistent with non‐specific abnormalities in 3/25 (12%). The latter included leucocytosis in three cats (WBC 4.88 × 10^9^ cells, RI: 6 to 15 × 10^9^ cells). Serum biochemistry was within normal limits in 19/25 (76%) cats, and consistent with non‐specific abnormalities in 6/25 (24%). The latter included hyperglobulinaemia in two cats (56 and 62 g/L, RI: 26 to 54 g/L), increased ALP in three cats (77, 108, 84 U/L; RI: 0 to 50 g/L), increased ALT in one cat (84 U/L, RI: 0 to 60) and increased BUN (15.9 mmoL/L, RI: 4 to 12 mmol/L) and creatinine (284 μmol/L, RI: 80 to 180 μmol/L). Total thyroxine was assessed only in 13/25 (52%) and it was within normal limits. The median age of the cats in which total thyroxine was not checked was 4.4 years (range, 0.5 to 12.3 years; IQR, 6.3 years), all of them had normal haematology and serum biochemistry, and none of them had signs of hyperthyroidism. Vitamin B12 was only checked in two cats and it was within normal limits. Infectious disease tests were done in 5/25 (20%) cats including serology (IFA) for *Toxoplasma gondii* IgG and IgM (*n* = 3), serology (ELISA snap test) for FIV and FeLV (*n* = 5) and PCR in the cerebrospinal fluid (CSF) for *T. gondii*, *Cryptococcus* spp. and Feline Coronavirus (*n* = 1), that was all negative. Magnetic resonance imaging of the head was performed in 14/25 (56%) cats, one of which included also the whole vertebral column, and CT of the head was performed in 1/25 (4%) cats, and they were all considered unremarkable. In one case with T3–L3 myelopathy, MRI revealed thoracic stenosis consisting of hemivertebra at T7 and T7–T8 intervertebral disc protrusion with associated focal T2W intramedullary hyperintensity at this level consistent with oedema or gliosis. Cerebrospinal fluid analysis was performed in 13/25 (52%) cats, and it was within normal limits. There was no serology test for autoantibodies against gliadin IgG or transglutaminase‐2 IgA done for any of the cats in this study. All cats were presumptively diagnosed with PxD.

Treatment was attempted in 11/25 (44%) cats. This included a gluten‐free diet trial (6/11; 55%), levetiracetam, phenobarbital or gabapentin (2/11; 18% each). Strict gluten‐free diet trial decreased episodes of PxD in 4/6 (66.7%) cats at a median follow‐up period of 3.5 months (range 3 to 5 months), during which the diet was administered. Levetiracetam (30 mg/kg PO q8h) ceased the episodes completely in a cat at a 7‐month follow‐up. In the other cat, levetiracetam (20 mg/kg PO q8h) decreased the episodes for a 1‐year follow‐up. After that, episodes increased, and the owners elected to discontinue it. Phenobarbital (2.5 mg/kg PO q12h) did not alter the frequency of the episodes in two cats at a follow‐up of 3 months and 6 months, respectively. Gabapentin (10 mg/kg PO q8h) did not alter the episodes frequency in two cats at a 2‐month follow‐up. Based on treatment response, most cats (21/25; 84%) were presumptively diagnosed with idiopathic paroxysmal non‐kinesigenic dyskinesia.

In 4/25 (16%), cats paroxysmal gluten‐sensitive dyskinesia was suspected. One (1/4; 25%) of them was one of the few cats that presented with gastrointestinal signs (vomiting). Two of these cats had multiple episodes per day, one cat had five episodes and one cat had two episodes both within a 5‐month period. In these cats, improvement on a gluten‐free diet consisted of episode decrease from daily to fortnight in one cat and to every 2 weeks in another cat, and episode cessation in two cats during the treatment period.

## DISCUSSION

This is the first study to describe idiopathic non‐kinesigenic PxD in a diverse population of cats, providing additional information about the semiological phenotype of this disease in cats. This study also establishes the presence of PxD in multiple cat breeds other than Sphynx. This study also describes a few cases of cats with presumptive gluten‐sensitive PxD.

Identification of PxD can be mainly achieved by evaluation of the paroxysmal episodes in situ or through a video footage. Presence of advanced technology (*e.g*. smartphones) provides an easy, quick and inexpensive means of recording paroxysmal events (Matz et al., 2025; Packer et al., 2015). These episodes probably occurred in cats in the past. However, the previous lack of available technology to record these events, possibly led to a misdiagnosis as focal epileptic seizures.

Paroxysmal dyskinesia in cats seems to be an early‐onset disease as it manifested at a median age of 3.5 years in multiple breeds, similarly to what was reported previously in Sphynx cats (James et al., 2022). It seems that PxD is not a unique characteristic of purebreds (*e.g*. Sphynx), as most cats in our study were DSH. Additionally, accompanying gastrointestinal signs were uncommon in cats, in contrast to those previously described in dogs with PxD (Whittaker et al., 2022).

Phenotype of PxD in cats overall simulates the general patterns reported in dogs, primarily characterised by shifting limb dystonia, occasionally accompanied by head and neck (cervical dystonia), and trunk (kyphosis) dystonia. Interestingly, some unique features for cats were observed that could help characterise the clinical recognition of PxD in this species. During an episode, 44% of cats display a crawling gait, characterised by walking on all four limbs close to the ground. Almost one out of four cats also exhibited kneading (‘making biscuits’ movement), that is, a flexion and extension of the digits of the thoracic limb paws, while the thoracic limbs were dystonically extended. An additional observation was that during the dystonia, almost one out of two cats were adopting a bradykinetic (slow‐motion) gait, frequently followed by involuntary crossing of the thoracic limbs resembling proprioceptive ataxia. The latter is probably a result of limb dystonia forcing the thoracic limbs to cross over the midline. Of note, in human neurology, movement slowness is called bradykinesia and is traditionally linked to hypokinetic movement disorders and specifically Parkinson's disease. However, bradykinesia as a clinical sign can also be uncommonly seen in hyperkinetic movement disorders such as paroxysmal dyskinesia (Bologna et al., 2023). Bradykinesia has been already reported as a characteristic sign of PxD in Sphynx cats (James et al., 2022) and cats with hyperthyroid‐associated PxD (Espinosa et al., 2026), and it is not a sign seen in paroxysmal dyskinesia of dogs (Mandigers et al., 2024).

Moreover, cervical dystonia was present in almost 1/4 of cats. Dystonic head tremor was present only in 12% of cats with PxD in our study, and appears to be a rare finding in cats with PxD (Espinosa et al., 2026; James et al., 2022), in contrast to dogs that have a high prevalence (44%) (Liatis & De Decker, 2023). Another unique feature of cats with PxD was the tail dystonia (flexed or upright extended) observed in our population and in cats with hyperthyroid‐associated PxD (Espinosa et al., 2026), which has not been reported in dogs.

An interesting point acquired from the data is that in 1/4 of the population, PxD can manifest as sporadic clusters followed by long episodes of paucity. The reason for this presentation is not yet understood, and further research is warranted to shed light on factors associated with seasonality or environmental triggers of PxD in cats.

Classification of PxD in dogs and cats as kinesigenic or non‐kinesigenic can be challenging. The most common type of PxD in dogs is non‐kinesigenic (Mandigers et al., 2024). Similar, based exclusively on history in our study, there were no cats in which abrupt limb movements triggered PxD and most cats shared similar characteristics with non‐kinesigenic PxD. In contrast, PxD in 30% of Sphynx reported previously was triggered by sudden movement, excitement or stress and some were considered kinesigenic PxD (James et al., 2022).

Additionally, a small proportion of cats in this study had a dramatic response to a diet trial with gluten‐free diet, showing a marked reduction in the episode frequency. Whereas spontaneous improvement can occur in feline cases of PxD (James et al., 2022), the rapid response to the diet change supported a gluten‐sensitive PxD. Whereas gluten‐sensitive PxD has been well recognised in multiple breeds of dogs (Rogers et al., 2023) and has not been previously reported in cats.

Routine metabolic screening (electrolytes and glucose) was performed in all cats, and the results were normal and thus not indicative of major metabolic disease. However, the thyroid profile was assessed only in half of the cat population of this study, and therefore, a hyperthyroidism‐associated paroxysmal dyskinesia that has been recently described in cats cannot be ruled out completely (Espinosa et al., 2026). Nevertheless, this population of cats in which the thyroid profile was not assessed was young, had no signs of hyperthyroidism and had normal haematology and serum biochemistry, and therefore the chances of missing hyperthyroidism were considered slim. Additionally, other biomarkers such as vitamin B12 that have been associated with dystonia in humans (De Souza & Moloi, 2014) were not tested at all.

When performed, diagnostic investigations including MRI of the head and CSF analysis, reportedly yielded no significant abnormal findings, albeit this work‐up was inconsistent and not performed in every case. These findings are consistent with previous reports in both species (James et al., 2022; Mandigers et al., 2024) where diagnostic evaluations are typically normal. Hence, similar to dogs (Mandigers et al., 2024), PxD in cats was considered most likely an idiopathic disorder. No genetic mutations associated with PxD in cats have been found yet. However, the over‐representation of specific breeds (*e.g*. Sphynx) (James et al., 2022), raises a suspicion of a genetic background in these breeds. A small part of the population in this study (16%) responded to the gluten‐free diet trial, and therefore their PxD could be considered dietary in origin, as previously described in dogs (Rogers et al., 2023). All in all, regardless of the phenotypic diagnosis, further investigations are warranted to identify the underlying aetiology of the PxD (metabolic, structural, pharmacological, *etc*.).

In our study, half of the cat population did not receive treatment. The other half received a gluten‐free diet trial and 66.7% of them responded by decreasing the episode frequency at a median time of follow‐up of 3.5 months during which treatment was administered. Also, in the limited number of cases in which antiseizure medications were administered, levetiracetam appeared to be effective in both cases in which it was used for a follow‐up period of 7 to 12 months, whereas gabapentin and phenobarbital did not reduce episode frequency. Treatment was not pursued in most of the Sphynx cats previously described (James et al., 2022). However, two cats who received acetazolamide had a reduction in the number of episodes, although an extended follow‐up was not available for them. Acetazolamide was not administered in our study.

Limitations of our study included its retrospective nature, the lack of homogeneous investigations, lack of some specific investigations in each case (thyroid profile, vitamin B12 and folate, MRI scan of the head and CSF analysis), complete lack of electroencephalography in any case, and lack of a homogeneous treatment protocol. In some cases, dietary or hyperthyroid‐associated or other structural PxD cannot be fully excluded based on provided diagnostic investigations. In presumptive paroxysmal gluten‐sensitive dyskinesia, no antigluten antibody serology has been done and the period of treatment and follow‐up was short.

In conclusion, PxD occurs in multiple cat breeds. Idiopathic paroxysmal non‐kinesigenic dyskinesia was the most common type of PxD; however, some cats manifested presumptive paroxysmal gluten‐sensitive dyskinesia.

Idiopathic PxD in cats has some distinctive clinical features compared to dogs, such as crawling, crossing of the thoracic limbs resembling proprioceptive ataxia, bradykinesia (slow‐motion movements), ‘kneading’ of the digits or tail dystonia, making the feline phenotype of PxD unique.

### Author contributions

**T. Liatis:** Conceptualization; investigation; writing – original draft; methodology; writing – review and editing; project administration; resources; visualization. **C. Maeso:** Conceptualization; investigation; writing – review and editing; methodology; visualization. **A. De Stefani:** Writing – review and editing; supervision. **A. Pergande:** Investigation; writing – review and editing. **T. Elvira:** Investigation; writing – review and editing. **A. Suñol:** Conceptualization; investigation; writing – review and editing.

### Conflict of interest

The authors declared no potential conflicts of interest with respect to the research, authorship and/or publication of this article.

### Funding

The authors received no financial support for the research, authorship and/or publication of this article.

## Data Availability

The data that support the findings of this study are available from the corresponding author upon reasonable request.
